# Prediction of clinical progression in nervous system diseases: plasma glial fibrillary acidic protein (GFAP)

**DOI:** 10.1186/s40001-023-01631-4

**Published:** 2024-01-12

**Authors:** Xiaoxiao Zheng, Jingyao Yang, Yiwei Hou, Xinye Shi, Kangding Liu

**Affiliations:** 1 Department of Neurology, Neuroscience Center, The First Hospital of Jilin University, Xinmin Street 1#, Changchun, China; 2 Institute of Physiology, School of Basic Medical Sciences, Shanxi Medical University, Taiyuan, China; 3 Department of Cardiology, Shanxi Yingkang Yisheng General Hospital, Renmin North Road 5188#, Yuncheng, China

**Keywords:** GFAP, Astrocytes, GFAP astrocytopathy, Gliomas, Traumatic brain injury, Ischemic stroke, Alexander disease, Down syndrome, Creutzfeldt–Jakob disease, Alzheimer's disease

## Abstract

Glial fibrillary acidic protein (GFAP), an intracellular type III intermediate filament protein, provides structural support and maintains the mechanical integrity of astrocytes. It is predominantly found in the astrocytes which are the most abundant subtypes of glial cells in the brain and spinal cord. As a marker protein of astrocytes, GFAP may exert a variety of physiological effects in neurological diseases. For example, previous published literatures showed that autoimmune GFAP astrocytopathy is an inflammatory disease of the central nervous system (CNS). Moreover, the studies of GFAP in brain tumors mainly focus on the predictive value of tumor volume. Furthermore, using biomarkers in the early setting will lead to a simplified and standardized way to estimate the poor outcome in traumatic brain injury (TBI) and ischemic stroke. Recently, observational studies revealed that cerebrospinal fluid (CSF) GFAP, as a valuable potential diagnostic biomarker for neurosyphilis, had a sensitivity of 76.60% and specificity of 85.56%. The reason plasma GFAP could serve as a promising biomarker for diagnosis and prediction of Alzheimer's disease (AD) is that it effectively distinguished AD dementia from multiple neurodegenerative diseases and predicted the individual risk of AD progression. In addition, GFAP can be helpful in differentiating relapsing–remitting multiple sclerosis (RRMS) versus progressive MS (PMS). This review article aims to provide an overview of GFAP in the prediction of clinical progression in neuroinflammation, brain tumors, TBI, ischemic stroke, genetic disorders, neurodegeneration and other diseases in the CNS and to explore the potential therapeutic methods.

## Introduction

Glial fibrillary acidic protein (GFAP) is a specific astrocyte biomarker protein, which plays an important role in modulating the stability of cytoskeletal structure, maintaining mechanical potential of glial cells, and supporting the neighboring neurons [1, 2]. Increasing findings have suggested that GFAP has mainly been described in a variety of nervous system injuries and disorders. Recent research has revealed that astrocytes respond to an increased GFAP level after immune system diseases [3], tumors [4], trauma [5], ischemic [6] or infectious or genetic diseases [7] or neurodegenerative insult [8]. Here we elaborated the implications of GFAP in the pathological processes of diverse neurological diseases, aiming at exploring the on potential therapeutic strategies from the perspective of GFAP (Table 1).

**Table 1:** The applications of GFAP in diseases

Diseases	Research findings	GFAP applications in diseases	Animal models
Autoimmune GFAP astrocytopathy	The positive result of GFAP suggests this disorder, which may coexist with other antibodies (like NMDAR–IgG, AQP4–IgG) or meningitis (like TB)	Clinicians need to be aware of possible co-existence between GFAP astrocytopathy and other antibodies/meningitis	/
Gliomas	The more higher sGFAP level, the WHO grade of gliomas is more higher	sGFAP may be a rapid tool for the diagnosis and follow-up	/
TBI	GFAP reflects the disruption of astrocyte cytoskeleton and their activation in response to TBI	FDA has approved that a rapid blood test – GFAP/ UCH-L1 to aid the diagnosis of acute TII in mTBI patients	GFAP levels in serum and CSF both are elevated
Ischemic stroke	NIHSS at 24 h combined with either tau, NFL or GFAP at 48 h has an improved prediction	GFAP might be a key marker that can discriminate hemorrhagic stroke and ischemic stroke; GFAP in the early setting after endovascular treatment of stroke will be used as a simplified and standardized way to estimate the range of damaged nervous tissue	GFAP released within 3–4 h following hemorrhagic stroke, while it released within 24–48 h post injury in ischemic stroke
AxD	Abnormal RF accumulation because ofdisease-causing GFAP aggregate accumulation leads to astrocyte dysfunction; aggregations of GFAP are deleterious to astrocytes and thus lead to subsequent white matter degeneration	The presence of RF Is a hallmark feature of AxD, and identifying this pathology is key to diagnosis of this condition	Mice with GFAP knockout or GFAP point mutations display a mild phenotype (with strain-dependent deficits in cognition and RF, astrogliosis, increased seizure susceptibility in pathology but without motor deficits and leukodystrophy)
DS	The levels of GFAP increase and the GFAP-positive astrocytes proliferate in the brain of adult DS subjects	It provides a new insight into the plasticity potential of the brain by long-term voluntary running trains that positively affect the levels of GFAP and reduction of astrogliosis	Long-term voluntary running models reduce the number of GFAP-positive astrocytes and the levels of GFAP in the brain
AD	The concentrations of blood GFAP consistently increases in a stepwise pattern from preclinical AD, through prodromal AD to AD dementia compared to CU individuals; the higher levels of plasma GFAP, the more accurate predictive value for risk of AD progression	High GFAP level is associated with the poorer outcomes in AD	DMF can inhibit the immunoreactivity of GFAP also is inactivation of astrocytes
Neurosyphilis	The level of sGFAP parallels to the GFAP level in the CSF	A combination test of sGFAP, sNFL, and sUCH-L1 exhibits a specificity of 96.08% and a PPV of 94.60%	/
COVID-19	COV-Enc shows significantly higher CSF levels of glial-related markers such as GFAP, TREM2, and YKL-40 (*P* < 0.001) compared to HC, COV-Enc patients showed increased glial markers (GFAP, sTREM2, YKL-40) levels compared to ENC	GFAP has a significant predictive value in the prognosis of COVID-19 outcome	/
MS	GFAP is higher in MS patients than controls, GFAP levels are higher in PMS versus RRMS	GFAP can be helpful to define people whether in disease stage and in discriminating different subtypes	GFAP can be helpful to define people whether in disease stage and in discriminating different subtypes
Neuropsychiatric disorders	GFAP levels in children with autism are almost three times higher than in the group of children without autism	GFAP as biomarker protein for neuropsychiatric disorders; the higher GFAP concentration could be regarded as a pivotal role in improving behavioral response of neuropsychiatric disorders	The IS group showed significant reduction in the protein and mRNA levels of GFAP, whereas the IS + EE group cultures exhibited significant increase in the levels of these stem cell markers
Acute CO poisoning	The serum level of GFAP is significantly high in the NS group in comparison to the non-NS group	Initial GFAP protein level in the early identification of patients can predict the risk of developing NS after acute CO poisoning	/

*NMDAR* N-methyl-D-aspartate receptor, *IgG* immunoglobulin G, *AQP4* aquaporin-4, *TB* tuberculous, *sGFAP* GFAP in serum, *TBI* traumatic brain injury, *UCH-L1* ubiquitin carboxyl-terminal hydrolase L1, *mTBI* mild traumatic brain injury, *NFL* neurofilament light, *AxD* Alexander disease, *RF* Rosenthal fibers, *DS* Down syndrome, *AD* Alzheimer disease, *CU* cognitively unimpaired, *DMF* dimethyl fumarate, *sNFL* serum Neurofilament light, *sUCH-L1* serum ubiquitin carboxyl-terminal hydrolase L1, *PPV* positive predictive value, *COVID-19* coronavirus disease 2019, *COV-Enc* COVID-19-related encephalitis, *ENC* Encephalitis, *HC* healthy controls, *MS* multiple sclerosis, *PMS* progressive MS, *RRMS* relapsing–remitting MS, *IS* immobilization stress, *EE* enriched environment, *CO* carbon monoxide; *NS* neuropsychiatric sequelae; / not available

However, in vivo studies have been hampered by the difficulty in obtaining primary human astrocytes. Recently, to address the function of GFAP In vivo, the GFAP gene disrupted (GFAP^–^/^–^) in mice via targeted mutation in embryonic stem cells are ideal models used to investigate the functions of GFAP by researchers [9]. GFAP’s functional significance was easily explored through comprehensive analysis of the phenotypes of GFAP^–^/^–^ mice. The studies showed that mice with GFAP deficiency developed normally from production to adulthood and reproduced [9], but GFAP-negative astrocytes were completely lacking intermediate filaments, suggesting that losing GFAP is not compensated for other intermediate filament proteins [10].

Because of the complex molecular regulatory mechanisms between the astrocytes and GFAP and the lack of comprehensive biological roles of specific GFAP components, our understanding remains limited and more research is urgently needed to continue in this field. Therefore, pre-clinical and clinical studies are warranted to understand the role of GFAP in neurological diseases.

The purpose of the current review is to describe our current understanding of the functions of GFAP, summarize recent evidences highlighting that the clinical relevance of high GFAP expression in injuries or diseases in the central nervous system (CNS), before finally highlighting new literatures that might further advance current understanding of the potential therapeutic role of GFAP manipulation in neurological function restoration.

## Expression of GFAP in a variety of disorders

### Autoimmune GFAP astrocytopathy

Autoimmune GFAP astrocytopathy is an inflammatory disease of the nervous system complicated with CNS infectious diseases as well as associated with the occurrence of some tumors or autoimmune diseases or as a para-neoplastic disorder, with presence of GFAP immunoglobulin G (IgG) in the serum or cerebrospinal fluid (CSF) as a specific biomarker [11]. In the majority of cases, GFAP astrocytopathy coexists with many other antibodies involving N-methyl-D-aspartate receptor (NMDAR)–IgG, aquaporin-4 (AQP4)–IgG, antinuclear, anti-endothelial cell, anti-cardiolipin, anti-neutrophil cytoplasmic, anti-double-stranded DNA and other antibodies in serum [12, 13]. However, there are novel cases of patients with GFAP astrocytopathy complicated by CNS infection, such as tuberculous (TB) meningoencephalitis [12]. It had a variety of clinical manifestations predominantly affecting the white matter of the brain, spinal cord, optic nerves, cerebral cortex and even subpial regions. Below are its characteristics: (a) 30–40% of patients have symptoms suggestive of systemic infection before the onset of CNS symptoms, most common being cough, rhinorrhea and sore throat [14]. Neoplasm is seen in about 25% of cases, with ovarian teratoma being particularly common [15]. Autoimmune disorders like rheumatoid arthritis are found in about 20% of cases; (b) The incidence rate for males and females is approximately 1:1 [14]; (c) The clinical presentation is highly variable but most commonly is meningitis; other clinical features, including encephalitis, myelitis, seizures, psychiatric disorders, ataxia and tremor [3, 15]; (d) The studies of brain magnetic resonance imaging (MRI) revealed a characteristic pattern of linear perivascular enhancement in the cerebral white matter perpendicular to the ventricle, originating from GFAP-enriched periventricular areas [15]; (e) CSF analysis often showed inflammatory changes, with white blood cell pleocytosis, high protein content, and low to normal glucose [15, 16]; (f) The disease responds well to steroid treatment, but relapses occur following the reduction or withdrawal of prednisone [12]; (g) In pathological studies, extensive inflammation was encountered around microvessels, paralleling with the radial inflammatory changes seen in brain MRI [17].

#### GFAP astrocytopathy concurrent with meningitis induced by multiple bacterial pathogens

Prior studies have found that GFAP antibodies always coexist with AQP4 or oligoclonal bands (OB) antibody and relate with the occurrence of some tumors. However, little concurrency of GFAP astrocytopathy and CNS infection was reported. The first reported novel case of GFAP astrocytopathy in the literature complicated with TB meningoencephalitis was in 2013, whose metagenomic next-generation sequencing (mNGS) results of bronchoalveolar lavage fluid revealed Legionella pneumophila and mycobacteria. Then, mycobacterium TB was detected in CSF, and GFAP antibodies were also detected in CSF and serum. This patient’s symptoms improved following anti-TB and steroid combination therapy [12]. Subsequently, a case reported a 53-year-old woman, who was diagnosed with aseptic meningitis and positive GFAP antibody [11]. Therefore, clinicians need to be aware of possible co-existence between GFAP astrocytopathy and meningitis caused by multiple bacterial pathogens.

### GFAP in gliomas

Glioma represents the most frequent primary intracranial malignancy, accounting for 44% of all CNS tumors and 70% of malignant primary brain tumors [4, 18]. Characterized by poorly differentiated neoplastic astrocytes and highly invasive behavior towards surrounding tissues, glioblastoma (GBM) is the most malignant form of all astrocytic tumors and the most common brain tumors in adults [4]. As the most lethal entity, GBM responds badly to current conventional cancer treatments, with 5-year survival of 2.7% [19]. Despite recent advances have been gotten in therapeutic strategies, the prognosis for patients harboring GBM remains undesirable with a median survival period of less than 18 months [4]. Recently, serum GFAP (sGFAP) was shown to be closely linked with glioma, but additional studies are necessary to fully explore the potential of sGFAP as a rapid tool for the diagnosis and follow-up of glioma and definite acting mechanism of GFAP in GBM [20].

#### GFAP expression has an inverse relation to the proliferation in vitro gliomas

Early studies have found that the content of GFAP is associated with the mitotic phase of the dividing cells. Interestingly, GFAP showed in an average low level when cells entering mitoses in the soma, progressively increased as the subsequent phases of mitosis progressed, reaching its highest levels during telophase and cytokinesis [21]. GFAP-deficient mice by gene targeting were generated for the study of the biological functions of GFAP. Pekny and his colleagues observed that primary cultures of GFAP − / − astrocytes exhibited increased proportion suggesting the loss of GFAP expression frequently observed in WHO high-grade gliomas could be a key step towards progression to a more rapidly growing and malignant phenotype in brain tumors [22].

Another study also indicated that sGFAP levels were significantly higher in 62.7% of all WHO grade-IV patients compared to 12.7% of healthy controls (HC, *P* < 0.05), which was in accordance with the previous data analysis in animal models. Moreover, sGFAP showed an average median difference of 0.15 ng/mL in WHO grade-IV gliomas compared to HC (0.04 versus 0.25, *P* < 0.01). They also found evidences for sGFAP levels as a predictor of tumor volume not patient outcome [20]. However, further research is needed to fully confirm the effectiveness of sGFAP as a tool for the diagnosis and follow-up of WHO grade-IV glioma.

#### The GFAP suppressors can inhibit GBM progression

**Fig. 1:**
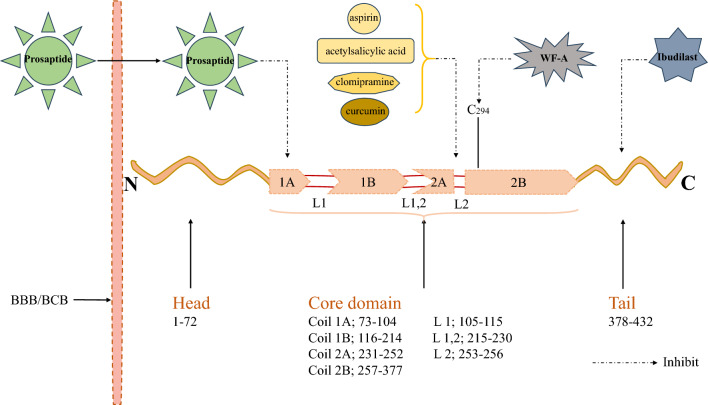
Linear structure, functional domains and key modifications of GFAP-α. GFAP-α is the most abundant subtype, which contains the head, core domain, and tail. The core domain is composed of the coil domain (**1A**, **1B**, **2A**, **2B**) and linker regions (L1, L1,2 and L2). The expression of GFAP protein is inhibited by a number of agents. Prosaptide, a peptide derived from a 14-amino-acid (Thr-D–Ala–Leu–Ile–Asp–Asn–Asn–Ala–Thr–Glu–Glu–Ile–Leu–Tyr) neurotrophic sequence of human glycoprotein prosaposin, which can cross BBB or BCB to exert its GFAP-suppression effects and thereby exert its therapeutic effects. In addition, WF-A, a steroidal lactone isolated from Ayurvedic medicine Winter cherry, also have been studied that it can inhibit GFAP and the related IF protein vimentin via the covalent modification of the single Cys-294 of GFAP. And then, Ibudilast, a board-spectrum PDE inhibitor, was unexpectedly found to also inhibit methamphetamine-induced GFAP upregulation and gliosis. Furthermore, a number of drug-like agents have reported to have suppressed effects either in GFAP protein expression or gliosis induction, varying from aspirin/acetylsalicylic acid, clomipramine to curcumin (unknown exact mechanism). *L1* Linker 1; *BBB* blood brain barrier, *BCB* blood cerebrospinal fluid barrier, *WF-A* Withaferin A, *IF* intrinsic factor, *PDE* Phosphodiesterase

As mentioned above, GFAP plays a vital role in the proliferation and invasion of tumors [23]. GFAP-α, the most abundant subtype of GFAP, contains the N-terminal head, central core domain and C-terminal tail. The central core domain is composed of coil domain (1A, 1B, 2A, 2B) and linker regions (L 1, L 1,2 and L 2) [24] (Fig. 1). A great deal of therapeutic agents can considerably suppress the expression of GFAP or the function of glia cells via acting their specific areas to achieve the aim of monitoring or therapy (Table 2). Taylor et al. described a novel compound Prosaptide, a peptide derived from a 14-amino-acid (Thr–D-Ala–Leu–Ile–Asp–Asn–Asn–Ala–Thr–Glu–Glu–Ile–Leu–Tyr) neurotrophic sequence of human glycoprotein prosaposin, which can cross blood brain barrier (BBB) or blood–CSF barrier to exert its GFAP-suppression effects and thereby exert its therapeutic effects by combing with 1A of core domain [23, 25]. They examined the bioactivity of five peptidomimetics (Prosaptides D1–D5) and concluded that four retro-inverso peptidomimetics (Prosaptides D2–D5) retained bioactivity in neurite outgrowth except for inactive Prosaptide D1. Especially, (125)I-Prosaptide D4 remained intact for 60 min after intravenous injection and was transported into brain or serum across the BBB [25]. In addition, Withaferin A (WF-A), a steroidal lactone isolated from Ayurvedic medicine Winter cherry, also has been found to be a GFAP suppressor [26]. Several studies have demonstrated that WF-A can inhibit GFAP and the related intrinsic factor (IF) protein vimentin via the covalent modification of the single Cys-294 of 2B of GFAP and a homologous cysteine residue in vimentin protein, respectively. Here, Bargagna-Mohan et al. developed an alkali ocular injury model in mouse, whose predominant pathological change is reactive Müller cell gliosis characterized by the overexpression of the GFAP and vimentin, illustrating GFAP/vimentin targeting molecule WFA is a novel chemical probe of GFAP. In this study, WFA resulted in cell cycle G0/G1 arrest by binding to and down-regulating soluble vimentin and GFAP expression [27]. Thus, WF-A is BBB-permeable, leading to attenuated GFAP levels and activated glial cell. Ibudilast, a board-spectrum phosphodiesterase (PDE) inhibitor, was unexpectedly found to also inhibit methamphetamine-induced GFAP upregulation and gliosis by acting with C tail of GFAP [28]. Surprisingly, a number of drug-like agents have reported to have suppressed effects either in GFAP protein expression or gliosis induction, varying from aspirin/acetylsalicylic acid [29], clomipramine [30] to curcumin [31]. The specific action sites of the above-mentioned drugs are detailed summarized in Fig. 1.

**Table 2:** GFAP suppressors

GFAP suppressors			
Agents	Chemical essence	Site of action	Reference(s)
Prosaptide	A peptide derived from a 14-amino-acid neurotrophic sequence of human glycoprotein prosaposin	Precise site of action is unknown (Down-regulation of GFAP expression)	[23, 25]
WF-A	A steroidal lactone isolated from Ayurvedic medicine (Winter cherry)	Covalent modification of the single Cys-294 of GFAP(Inhibit GFAP and the related IF)	[26, 27]
Ibudilast (AV411)	A board-spectrum PDE inhibitor	Precise site of action is unknown (Inhibit methamphetamine-induced GFAP upregulatio))	[28]
aspirin/acetylsalicylic acid	Cox-2 inhibitor	Precise site of action is unknown (Down-regulation of GFAP expression)	[29]
clomipramine	Tricyclic antidepressant	Precise site of action is unknown (Down-regulation of GFAP expression)	[30]
curcumin	Extracted from turmeric	Precise site of action is unknown (Down-regulation of GFAP expression)	[31]

*WF-A* Withaferin A, *IF* intrinsic factor, *PDE* Phosphodiesterase, *Cox* cytochrome oxidase subunit

Although the exact mechanism of how these agents decreased expression of GFAP is presently unknown, they still might provide useful models for us to study the role of GFAP in neuro-disease models. It is of necessity to find druggable molecules that can suppress GFAP expression or can even specifically reverse GFAP aggregate formation.

### GFAP in traumatic brain injury (TBI)

TBI can be roughly classified into two main groups: primary injury caused by the initial direct mechanical impact to the head, such as brain contusion, diffuse axonal injury, skull fracture and vascular injury, and secondary injury featured by complications, such as brain herniation, chronic traumatic encephalopathy (CTE) and diffuse cerebral edema [32]. TBI is a medical and economic burden for families, communities, and health-care systems globally that has a yearly incidence of 60 million people accounting for approximately 2% worldwide [33, 34]. One reason current diagnosis methods fail to meet the expectation of TBI patients is the high cost and radiation exposure of TBI-head computerized tomography (CT). A rapid, portable, accurate and more secure way of diagnosing has the potential to make patients have good compliance and reduce wait time for a head CT scan without these drawbacks.

#### A rapid GFAP/ubiquitin carboxyl-terminal hydrolase L1 (GFAP/UCH-L1) test for the prediction of mild TBI (mTBI)

**Fig. 2:**
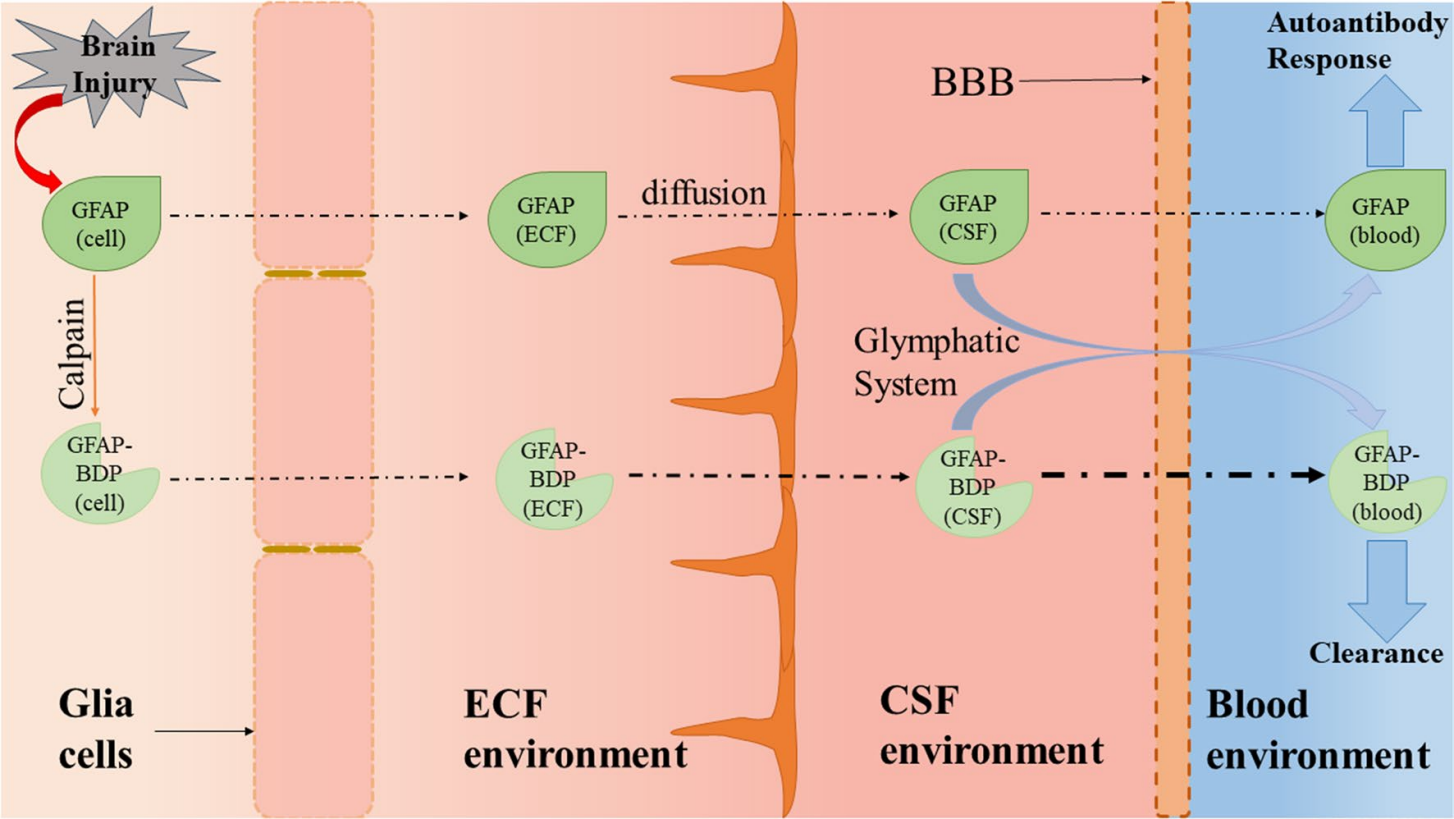
GFAP and GFAP–BDP released into ECF and blood when CNS injured. First, the figure shows GFAP–BDP is generated from GFAP when brain injured. Second, it describes how both GFAP and GFAP–BDP are released into ECF, and the later with a larger extent cascading response. Third, these two proteins diffused into the subarachnoid CSF. And then, GFAP/ GFAP–BDP either continue to follow the CSF flow through the ventricles or drain directly into the veins (Glymphatic System). Finally, these proteins enter the blood or circulation by diffusing through the BBB. Not only GFAP (blood) and GFAP–BDP (blood) can be served as biomarkers, but they can trigger autoantibody response as autoantigen or be cleared as foreign matters. *ECF* extracellular fluid, *CNS* central nervous system, *CSF* cerebrospinal fluid, *BBB* blood brain barrier

mTBI is common at an incidence exceeding 42 million people per year around the world, accounting for a large proportion (80–90%) of all head injury patients [35]. In animal models of TBI, GFAP levels in serum and CSF both are elevated [36]. GFAP is used as a biomarker for TBI, reflecting the disruption of astrocyte cytoskeleton and their reactiveness in response to TBI. FDA has approved that a rapid blood test combining measurements of both GFAP and ubiquitin carboxyl-terminal hydrolase L1 (GFAP/ UCH-L1) to aid the diagnosis of acute traumatic intracranial injury (TII) after mTBI patients in April, 2018 [37]. In addition, based on recent observations, GFAP–BDP (lysate of GFAP) also released into biofluids [extracellular fluid (ECF), CSF, blood], which was intimately tied to astrocyte damage or cell death after brain injury (Fig. 2) [38]. Then, we have reasons to speculate that the elevated levels of GFAP–BDP could be used to track the origin of astrocyte cell damage following brain trauma. Actually, this area has not been intensively studied and that must be verified with a larger sample size if it is used for novel therapy development.

### GFAP in ischemic stroke

Ischemic stroke, an acute cerebrovascular disease accompanied by poor cerebral circulation, neuronal damage, focal loss of neuronal function and other symptoms, is a leading cause of acquired, long term and physical disability in adults worldwide [39]. The primary approach and goal of therapy for stroke have focused on developing neuroprotective therapies. However, as the most abundant subtypes of glial cells, astrocytes are out number neurons by more than fivefold in the CNS [40]. It is controversial that reactive astrocytes after stroke play functional roles [41]. When CNS injured, astrocytes undergo important morphological modifications (such as hyperplasia, hypertrophy), also referred to as reactive Gliosis exhibiting increased intermediate filament proteins including GFAP, vimentin, and nestin [42], to form a physical barrier and functional wall around the damage area termed as “glial scar”. However, reactive astrocytes express a train of inhibitory molecules that are inhibitory for axonal regeneration in the “glial scar”. First, the “glial scar” may also separate non-injured tissue from the injured tissue, limiting the spread of tissue damage and uncontrolled inflammation [43]. Second, reactive astrocytes can play protective parts in the ischemic neurons by taking up excess glutamate [44] and producing neurotrophic factors [45]. Hence, it is the temporal and spatial conditions astrocytes are in that determine the reactivity of astrocytes and thus exert detrimental or beneficial effects. From that point of view, it may be a therapeutic target for neurorestorative strategies to manipulate the reactivity of astrocytes post stroke. Besides, prior studies have shown that GFAP released within 3–4 h following hemorrhagic stroke, while it released within 24–48 h post injury in ischemic stroke [6, 46, 47], strongly showing that GFAP might be a key marker that can discriminate these two.

#### High GFAP level is associated with the poorer outcomes in ischemic stroke

As a simplified and standardized way, the biomarkers in the early setting after endovascular treatment of stroke will be used to estimate the range of damaged nervous tissue, complement the possible clinical judgement and foresee further medical treatment or rehabilitation measures. In the study of Pujol-Calderon et al., every biomarker has its best time for prediction of poor outcome. It had a strong correlation between GFAP, neurofilament light (NFL), tau and patients’ outcomes, and also between infarction volumes and NIHSS at 24 h. Moreover, there was an improved prediction when NIHSS at 24 h combined with either tau, NFL or GFAP at 48 h [48]. In another study of Li et al., compared to patients only with basilar artery occlusion (BAO), the length of GFAP filaments had a significantly increased level in BAO with common carotid artery occlusion (BC) [49]. According to Deng’s research, inflammatory factors, such as TNF-α and IL-6, take part in excessive inflammation and oxidative cascade reactions when brain suffered ischemic injury [39]. Together with these previous proposals, we tentatively believe that GFAP was observed as a best biomarker for predicting poor outcome in ischemic stroke patients.

### GFAP in genetic disorders

#### Mutated GFAP proteins aggregate to produce astrocytic inclusions (called Rosenthal fibers, RF)

Alexander disease (AxD) is a rare genetic disorder, caused by missense mutations in the GFAP (the major intermediate filament protein in astrocytes) gene, resulting in astrocyte dysfunction, accumulation of GFAP aggregates and accumulation of stress protein aggregates (known as RF) in the brain. The presence of RF is a hallmark feature of AxD, and identifying this pathology is key to diagnosis of this condition [7]. Indeed, the first patient, a 15-month-old boy, was identified by William Stewart Alexander in 1949 [50]. Until 1964, Fried summarized five reported similar patients and named it “AxD” [7]. How various GFAP mutations are linked to pathology of AxD? In previous animal experiments, mice were designed to be GFAP knockout models or carry GFAP point mutations. However, the existing mouse models are not ideal, which display mild phenotypes with strain-dependent deficits in cognition and RF, astrogliosis, increased seizure susceptibility in pathology but without motor deficits and leukodystrophy, and are therefore suggested to be “gain-of-function” [23, 51]. Some researchers believe abnormal RF accumulation because of disease-causing GFAP aggregate accumulation leads to astrocyte dysfunction, while others believe that aggregations of GFAP are deleterious to astrocytes and thus lead to subsequent white matter degeneration pathology observed in AxD [52]. Interestingly, caspase-cleaved N-terminal GFAP fragment has the potential to promote GFAP aggregate formation [53].

#### Long-term voluntary running models for Down syndrome (DS) reduce the number of GFAP-positive astrocytes and the levels of GFAP in the brain

Previous studies have suggested the levels of GFAP increased [54] and the GFAP-positive astrocytes proliferated [55] in the brain of adult DS subjects. In addition, it was demonstrated that voluntary long-term running improved cognition and motor skills. Based on the above evidences, we hypothesized that long-lasting voluntary running could affect the brain levels of GFAP and astrocytes reactivity. With such questions, we reviewed the literatures and found that someone had proposed the same hypothesis and conducted experiments. In the experiments of Li et al., sedentary Ts65Dn mice served as controls. To determine whether long-term running may induce reactive astrogliosis in Ts65Dn animals, they measured the levels of GFAP in the brain of Ts65Dn mice by immunoblotting. It was showed that in comparison with controls, forced treadmill running models induced proliferation of astrocytes and showed an increased GFAP level in adult rats, which were forced to run on a treadmill for 30 min daily for up to six weeks [56]. In contrast, long-term voluntary running animals showed markedly reduced reactive astrogliosis and a lower GFAP level [57]. Therefore, studies have provided a new insight into the plasticity potential of the brain by long-term voluntary running trains that positively affect the levels of GFAP and reduction of astrogliosis.

#### Plasma GFAP predicts stroke in cerebral autosomal dominant arteriopathy with subcortical infarcts and leukoencephalopathy (CADASIL)

CADASIL is the most common hereditary cerebral small vessel disease caused by the *NOTCH3* gene mutations, characterized by migraine, stroke and vascular dementia in the patients [58]. In the study of Chen containing 63 CADASIL patients, they found that plasma GFAP was significantly elevated in the CADASIL patients compared with controls. Then, within a mean follow-up period of 3.1 ± 2.1 years, 6 patients (9.5%) had intracerebral hemorrhage (ICH) and higher GFAP (95% CI 1.21–6.53) predicted incident ICH [59]. Plasma GFAP may serve as a sensitive biomarker for CADASIL.

### GFAP in neurodegenerative diseases

The concentration of GFAP increased in the CSF of patients with neurodegenerative diseases, particularly Creutzfeldt–Jakob disease (CJD) and dementia [60]. The expression of GFAP was reported a significant (*P* < 0.05) decrease in the *B*. serrata extract containing boswellic acid (BSE)-treated groups [61]. In Long’s study recruited 818 GFAP–astrocytopathy individuals, 15.8% had dementia symptoms [13]. It seemed that GFAP antibodies were found to be a hallmark in patients with dementia according to findings of Barthel et al. [2].

#### High GFAP level is associated with the poorer outcomes in Alzheimer’s disease (AD)

AD is a progressive neurodegenerative disorder characterized by the accumulation of beta-amyloid and tau proteins in the brain. GFAP leaked into the CSF and blood may result in the neuroinflammatory reaction associated with AD, and is perceived as a biomarker of reactive astrogliosis [62]. In an investigating study of 818 patients, the concentrations of blood GFAP are reported to be consistently increased in a stepwise pattern from preclinical AD, through mild cognitive impairment (MCI) to AD dementia compared to cognitively unimpaired (CU) individuals. It not only could effectively distinguish AD from controls [area under the curve (AUC) > 0.97], and further distinguished preclinical (AUC > 0.89) and MCI (AUC > 0.85) from controls, but the higher levels of plasma GFAP, the more accurate predictive value for risk of AD progression [8]. Actually, plasma GFAP is useful both to be a diagnostic and predictive biomarker for AD, but more convinced evidences are required.

Likewise, in postmenopausal AD models (using D-galactose administered to ovariectomized rats), rats were grouped into sham-operated and ovariectomized/D-galactose groups that were either left untreated or treated with dimethyl fumarate (DMF) for 56 days starting three weeks after operations. The research finding that DMF can inhibit the immunoreactivity of GFAP as well as make astrocytes inactive, indicated GFAP strongly correlated to the progression of postmenopausal-associated dementia [63].

Caused by the triplication of the amyloid precursor protein (APP) encoded on chromosome 21.2, overexpression of APP lead to overproduction of amyloid-β (Aβ) peptide and increased deposition in the brain and thus to DS or AD [64]. Therefore, in a way, DS is assumed as a manifestation form of genetically determined AD. Similarly, the potential of plasma and CSF GFAP as a diagnostic and prognostic biomarker for AD and DS both were assessed. There was a longitudinal study using receiver operating characteristic curves (ROC) analyses to compare different plasma GFAP levels as diagnostic biomarkers in asymptomatic DS, symptomatic DS, autosomal dominant and sporadic AD. The sensitivity analysis showed that plasma GFAP was the plasma biomarker with the highest fold-change (AUC > 0.80) to discriminate symptomatic from asymptomatic participants. Notably, plasma GFAP was the earliest increased and the largest fold-changed indicator in the dementia stage, as well as the more large changes in autosomal dominant AD than in sporadic AD. When individuals stratified by amyloid PET or CSF Aβ42/40 ratio, concentrations of plasma GFAP could serve as a discriminated indicator of amyloid positive from amyloid negative DS patients. In addition, the concentrations of plasma GFAP in progressor significantly differed from non-progressors, and the difference also appeared in symptomatic groups vs asymptomatic groups [65].

Therefore, GFAP has emerged as a promising biomarker in dementia related to AD, but further evidence is required in relation to its utility as a biomarker of other kinds of dementia diseases.

### GFAP in neurological infectious diseases

Infectious diseases of the CNS usually were diagnosed by invasive operation lumbar puncture (LP), for one thing, it is not always well tolerated by patients, for another thing repetitive operation needs to be done aiming to clarify the efficiency of accepted treatment. To diagnose and monitor the prognosis of CNS infectious diseases, exploring non-invasive examinations or tests is of necessity.

#### The role of GFAP in neurosyphilis

Neurosyphilis is a clinical result of *Treponema pallidum* invading and causing various degrees of damage to the CNS [66, 67]. The diagnosis of it mostly depends on abnormal CSF results acquired from LP, mainly including a rapid plasma reagin (RPR) test and a Treponema pallidum particle agglutination test (TPPA) [68, 69]. Although it has been showed that using some found novel diagnostic biomarkers, such as C–X–C motif chemokine 13 [70, 71], CSF migration inhibitory factor [72], CSF soluble triggering receptor expressed on myeloid cells 2 [73], circulating brain-enriched GFAP, NFL, and UCH-L1 levels [74] in CSF, can assist to diagnose neurosyphilis, exploring reliable blood biomarker indicators is more convenient and acceptable to collect for diagnosing neurosyphilis. The current study revealed that patients with neurosyphilis had significantly higher sGFAP level compared to patients with uncomplicated syphilis and non-syphilis. In addition, the level of sGFAP paralleled to the GFAP level in the CSF, further suggesting that sGFAP has comparable to or even superior diagnostic performance than the CSF [75].

What’s more, as blood candidates for predicting the likelihood of neurosyphilis, the AUCs for sGFAP, serum NFL (sNFL), and serum UCH-L1 (sUCH-L1) were 0.86, 0.90 and 0.97, respectively (with sensitivities of 80.40–90.02%, with specificities of 78.43–80.39%) who are higher than that of serum RPR [76]. Besides, a combination of sGFAP, sNFL, and sUCH-L1 exhibited a specificity of 96.08% and a positive predictive value (PPV) of 94.60% while a single indicator with 10–20% missed or delayed diagnosis rate [75]. To improve the likelihood of detecting neurosyphilis avoiding LP, a testing format combined sGFAP, sNFL, and sUCH-L1 serving as a good entry point among patients without HIV, which can further improve the diagnostic sensitivity and specificity.

#### A strong correlation between increased GFAP level and coronavirus disease 2019 (COVID-19) patients with fatal outcome

COVID-19 patients with neurological symptoms have been reported frequently since 2019. De Lorenzo et al. collected clinical data of hospitalized COVID-19 patients, divided individuals into a group of patients with mild to moderate outcome and with severe even fatal outcome, performed ROC analyses using levels of sGFAP, sNFL and serum total tau (sT-tau), and concluded that levels of these three serum indicators all were elevated [77]. The performed ROC analysis showed that three biomarkers all had significant predictive values in the prognosis of COVID-19 outcome [77]. In another study of COVID-19 patients, Virhammar et al. extensively studied biomarkers in CSF for predicting the likelihood of neurological manifestations and disease severity resulting from COVID-19. Their report suggested that levels of GFAP, NFL protein, and T-tau in CSF commonly increased, and plasma levels of these biomarkers were parallel to CSF levels in COVID-19 patients with neurological symptoms [78]. Besides, researches among COVID-19 patients, there was an article comparing HC, COVID-19-related encephalitis (COV-Enc) and encephalitis (ENC) groups. Compared to the first two groups, COV-Enc showed significantly higher CSF levels of glial-related markers, such as GFAP, TREM2, and YKL-40 (*P* < 0.001). Compared to the latter two groups, COV-Enc patients showed increased glial markers (GFAP, sTREM2, YKL-40) levels [79]. In particular, elevated glial markers levels are indicative of early alterations of SARS-CoV-2 infection and may be valuable tools for monitoring disease severity.

### GFAP in other neurological diseases

#### GFAP is expressed in different stage of multiple sclerosis (MS) activity

MS is an autoimmune CNS disease, characterized by inflammation and demyelination. In MS [80], GFAP is expressed in reactive astrocytes within MS plaques, suggesting that GFAP plays a role in modulating astrocytic processes in response to the pathological changes associated with MS. Momtazmanesh et al. performed a meta-analysis including 4071 subjects to compare changes in CSF levels of neuronal and glial biomarkers between various variations of MS. This reported that CHI3L1, GFAP, NFL, S100B, and T-tau were higher in MS patients than in controls. In addition, GFAP levels were higher in progressive MS (PMS) versus relapsing–remitting MS (RRMS) [81]. Therefore, GFAP can be helpful to clarify people whether in the stage of disease and in discriminating different subtypes.

#### GFAP as biomarker protein for neuropsychiatric disorders

Initially, Ahlsén et al. analyzed GFAP levels in CSF of children and adolescents with autism and found that GFAP levels in children with autism were almost three times higher than in the group of children without autism in 1993 [82]. By comparison, another study has assessed the serum levels of GFAP in a Paediatric Acute-onset Neuropsychiatric Syndrome (PANS) cohort and 10 age-matched controls and mean GFAP concentrations did not differ with these two groups and no neurochemical evidence of neuronal injury or glial activation was found in PANS children [83]. In fact, this is an under-investigated area that could be exploited for monitoring the early development of PANS.

Similarly, the current study investigated the expression of GFAP in mice and rats with depressive-like phenotypes induced by exposure to various types of stress and the GFAP levels reduced [84, 85]. Accumulating evidence supports a key role of GFAP in detecting the possibility of major depressive disorder (MDD). The immobilization stress (IS) group showed significant reduction in the protein and mRNA levels of GFAP, whereas the IS + enriched environment (EE) group cultures exhibited significant increase in the levels of these stem cell markers [86]. The higher GFAP concentration could be regarded as a pivotal role in improving the behavioral response of rats.

The group difference was observed in plasma exosome concentrations got from schizophrenia patients compared to matched HC [87]. Considering small samples of this study groups, the finding result should be interpreted with caution and verified in larger cohorts.

#### GFAP can quantify the degree of neuronal damage of carbon monoxide (CO) poisoning

Acute CO poisoning is a frequent cause of acute intoxication worldwide with a high level of mortality and high risk of developing persistent or delayed neuropsychiatric sequelae (NS) occurred in up to 50% of patients. In comparison to the non-NS group, the serum level of GFAP was significantly high in the NS group with 95.24% sensitivity and 69.23% specificity at a cutoff value of 2.8 ng/mL of ROC [88], which in agreement with previous studies. In a study by Ghorbani et al., significantly higher GFAP levels were found after exposure to CO compared with the control group [89]. Akdemir et al. reported that mean GFAP concentrations differ between patients and controls [90]. Above data demonstrated a vital role of initial GFAP protein level in the early identification of patients at risk of developing NS after acute CO poisoning and in deciding treatment plans and thus improving quality of care.

## Detection methods for GFAP levels

**Fig. 3:**
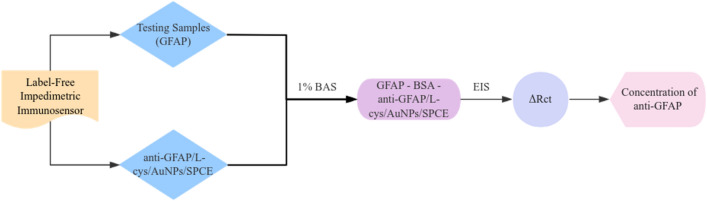
Label-free impedimetric immunosensor for GFAP detection. L-cys was explored as a substrate for the immunization of GFAP antibodies because it enables the guided and stable immobilization of GFAP antibodies. In addition, it served as a linker between GFAP antibodies and Au NPs/SPCE. Additionally, the combination of L-cys and Au NPs is likely to exhibit high effective surface area and stability. Furthermore, 1% BSA was adsorbed on the surface to block the –COOH groups and the non-interacting –NH_2_ groups of the GFAP antibody, and prevent non-specific binding. In this label-free impedance immunosensor, testing samples can immobilize anti-GFAP antibodies via covalent attachment onto L-cys/ Au NPs that were modified with anti-GFAP/L-cys/Au Nps/SPCE for the detection of GFAP. Finally, using EIS to measure the ΔRct, which had a linear correlation with GFAP concentration. BSA was adsorbed on the surface to block the activated carboxyl group and prevent non-specific binding. *L-cys* L-cysteine, *GFAP* glial fibrillary acidic protein, *Au NPs* gold nanoparticles, *SPCE* screen printed electrode, *EIS* electrochemical impedance spectroscopy, *ΔRct* transfer resistance differences

Above all, GFAP has a good diagnostic ability to predict a variety of disorders. Therefore, there summarizes assays that may be utilized from patients’ samples to monitor GFAP levels. General measurements include ELISA [91], electrochemiluminescent (ECL) [92], dissociation-enhanced lanthanide fluorescence immunoassay (DELFIA) system [93], and time-resolved fluorescent lateral flow immunoassay (TRF–LFIA) [94]. In addition, there are a few current examples, that have been published in recent works, such as chemiluminometric immunoassay [95], electrochemical immunosensors [96] and label-free impedimetric immunosensor. We mainly introduced the L-cysteine (L-cys) functionalized gold nanoparticles (AuNPs)-based screen printed electrode (SPCE) immunosensor for GFAP detection. L-cys enable the guided and stable immobilization of GFAP antibodies, thus resulting in its linker role between GFAP antibodies and Au NPs/SPCE. Moreover, AuNPs can function as electron donors to enhance electron transfer and increase conductivity. In this label-free impedance immunosensor, testing samples can immobilize anti-GFAP antibodies via covalent attachment onto L-cys/AuNPs that were modified with anti-GFAP/L-cys/AuNps/SPCE for the detection of GFAP. Finally, using electrochemical impedance spectroscopy (EIS) to measure the transfer resistance differences (ΔRct), which had a linear correlation with GFAP concentration (Fig. 3) [97].

## Conclusion

Physiological knowledge on the functions of GFAP and various roles of GFAP expression in predicting likelihood of disorders have recently increased, while these are just drops in the bucket and further research is necessary to be explored. As a highly specific marker of astrocyte activation and a significant structural element of astrocytes, we show the high prognostic value and clinical utility of GFAP as an ideal candidate to select high-risk individuals and further prevent early in clinical trials or treatment timely in patients. However, it remains unclear whether GFAP is simply a marker of a more generalized astrocyte activation or even an initial direct driver of the inflammatory cascading responses. Taken together, GFAP is a potential diagnostic approach and therapeutic target for future strategies, as well as is of significant interest to clinicians and researchers alike. Further efforts are warranted to fully elucidate the pathogenic mechanism of GFAP, which is crucial to our better understanding of GFAP-related diseases, and may further open new experimental and therapeutic avenues.

## Data Availability

Not applicable.

## Abbreviations

GFAP: Glial fibrillary acidic protein
CNS: Central nervous system
IgG: Immunoglobulin G
CSF: Cerebrospinal fluid
NMDAR: N-methyl-D-aspartate receptor
AQP4: Aquaporin-4
TB: Tuberculous
MRI: Magnetic resonance imaging
OB: Oligoclonal bands
mNGS: Metagenomic next-generation sequencing
GBM: Glioblastoma
sGFAP: GFAP in serum
HC: Healthy controls
BBB: Blood brain barrier
WF-A: Withaferin A
IF: Intrinsic factor
PDE: Phosphodiesterase
TBI: Traumatic brain injury
CTE: Chronic traumatic encephalopathy
CT: Computerized tomography
mTBI: Mild TBI
UCH-L1: Ubiquitin carboxyl-terminal hydrolase L1
TII: Traumatic intracranial injury
ECF: Extracellular fluid
NFL: Neurofilament light
BAO: Basilar artery occlusion
BC: BAO with common carotid artery occlusion
RF: Rosenthal fibers
AxD: Alexander disease
DS: Down syndrome
ICH: Intracerebral hemorrhage
CJD: Creutzfeldt–Jakob disease
BSE: *B*. Serrata extract containing boswellic acid
AD: Alzheimer's disease
CU: Cognitively unimpaired
AUC: Area under the curve
DMF: Dimethyl fumarate
APP: Amyloid precursor protein
Aβ: Amyloid-β
ROC: Receiver operating characteristic curves
LP: Lumbar puncture
RPR: Rapid plasma regain
TPPA: Treponema pallidum particle agglutination test
PPV: Positive predictive value
COVID-19: Coronavirus disease 2019
sT-tau: Serum total tau
COV-Enc: COVID-19-related encephalitis
ENC: Encephalitis
MS: Multiple sclerosis
PMS: Progressive MS
RRMS: Relapsing–remitting MS
PANS: Paediatric Acute-onset Neuropsychiatric Syndrome
MDD: Major depressive disorder
IS: Immobilization stress
EE: Enriched environment
CO: Carbon monoxide
NS: Neuropsychiatric sequelae
ECL: Electrochemiluminescent
DELFIA: Dissociation-enhanced lanthanide fluorescence immunoassay
TRF-LFIA: Time-resolved fluorescent lateral flow immunoassay
L-cys: L-cysteine
Au NPs: Gold nanoparticles
SPCE: Based screen printed electrode
EIS: Electrochemical impedance spectroscopy
ΔRct: Resistance differences
